# Effects of Hydration and Temperature on the Microstructure and Transport Properties of Nafion Polyelectrolyte Membrane: A Molecular Dynamics Simulation

**DOI:** 10.3390/membranes11090695

**Published:** 2021-09-08

**Authors:** Guoling Zhang, Guogang Yang, Shian Li, Qiuwan Shen, Hao Wang, Zheng Li, Yang Zhou, Weiqiang Ye

**Affiliations:** 1 Marine Engineering College, Dalian Maritime University, Dalian 116026, China; zhangguoling@dlmu.edu.cn (G.Z.); shenqiuwan@dlmu.edu.cn (Q.S.); whdlmu@dlmu.edu.cn (H.W.); li.zheng@dlmu.edu.cn (Z.L.); brave@dlmu.edu.cn (Y.Z.); 2 School of Marine Engineering, Guangzhou Maritime University, Guangzhou 510725, China; ywqgmc@sina.com

**Keywords:** microstructure, Nafion, hydration, temperature, molecular dynamics simulation

## Abstract

To investigate the effects of temperature and hydration on the microstructure of polymer electrolyte membrane and the transport of water molecules and hydronium ions, molecular dynamics simulations are performed on Nafion 117 for a series of water contents at different temperatures. The interactions among the sulfonate groups, hydronium ions, and water molecules are studied according to the analysis of radial distribution functions and coordination numbers. The sizes and connectivity of water clusters are also discussed, and it is found that the hydration level plays a key role in the phase separation of the membrane. However, the effect of the temperature is slight. When the water content increases from 3.5 to 16, the size of water clusters in the membrane increases, and the clusters connect to each other to form continuous channels for diffusion of water molecules and hydronium ions. The diffusion coefficients are estimated by studying the mean square displacements. The results show that the diffusion of water molecules and hydronium ions are both enhanced by the increase of the temperature and hydration level. Furthermore, the diffusion coefficient of water molecules is always much larger than that of hydronium ions. However, the ratio of the diffusion coefficient of water molecules to that of hydronium ions decreases with the increase of water content.

## 1. Introduction

Polymer electrolyte membrane fuel cells (PEMFCs) have received particular attention, due to their renewable energy, zero emissions, high power density, high efficiency, low operating temperature and low noise advantages [1,2]. The polymer electrolyte membrane (PEM) is the core of the PEMFC, which plays an important role in transporting protons and blocking hydrogen and oxygen. PEM should have the characteristics of proton conductivity and good mechanical stability. Nafion is the most widely used polymer electrolyte membrane [3,4]. The chemical structure of Nafion consists of a hydrophobic polytetrafluoroethylene backbone and the hydrophilic perfluorosulfonic acid side chains, the hydrophobic backbone exhibits good mechanical, chemical and thermal stability, while the hydrophilic groups are used to transport protons. In the presence of water, the microstructure of Nafion facilitates phase separation, thus the hydrophilic clusters will be embedded in the hydrophobic phase [5]. To investigate the properties of PEMs, especially Nafion, a lot of experiments [6,7,8,9,10] have been undertaken. However, it is not easy to provide a molecular-scale view of the phase separation and the transmission in the system.

Molecular dynamics (MD) simulations are ideal methods to investigate the microstructures and the physical/chemical properties of PEMs. Bahlakeh et al. [11] investigated the effects of methanol solvent on the microstructure and the diffusion properties of SPPO membranes using molecular dynamics simulations. They found that phase separation of the membrane on the uptake of methanol. In addition, methanol is more difficult to transport in SPPO compared with Nafion. Zheng et al. [12] discussed the dynamic properties and thermal properties of three different membranes (Dow, Nafion and Aciplex) using MD simulations. They reported that Aciplex membrane presents a better transmission performance and a better thermal property. Yana et al. [13] interpreted the difference of diffusivity between Krytox-Silica hybrid Nafion and pure Nafion using MD simulations. They found that adding Krytox-Silica to the Nafion did not affect the diffusion coefficients of water molecules and hydronium ions greatly. Devanathan et al. [14] performed MD simulations to clarify the difference between phenylated sulfonated poly (ether ether ketone ketone) (Ph-SPEEKK) membrane and Nafion membrane in terms of morphology and transmission performance at 360 K for a series of water contents. The results showed that the average pore diameter in Ph-SPEEKK is smaller, compared with Nafion. The diffusion of water and hydronium is slower in Ph-SPEEKK at comparable hydration levels. Rao et al. [15] performed MD simulations on Nafion to elucidate the effect of crosslinking formation on proton diffusivity in PEM, and they reported that the proton diffusion coefficients increase at first and then decrease with the increase in crosslinking number. Rujie et al. [16] studied the structure-property relationship of SPEEK and Nafion membranes at the molecular level. Their results of MD simulations explained why the SPEEK membranes present a lower proton conductivity than Nafion membranes. MD simulations have also been performed to study the effects of the degree of deprotonation and water content on the internal structure and vehicular transport inside the Nafion membranes [17]. The temperature effect on the diffusion processes of water and proton in the Nafion 117 membranes was investigated using MD simulations by Lei et al. [18]. Cui et al. [19,20] performed MD simulations to study the effects of the length of the side chain and the water contents on the hydrated morphology and hydronium ion diffusion in perfluorosulfonic acid membranes at a temperature of 300 K. Jang et al. [21] investigated the effects of the monomeric sequence of the chain on the nanophase-segregation structure and transport in hydrated Nafion using MD simulations. Chen et al. [22] studied the effects of temperature and water content on the thermal conductivity of Nafion 117 using MD simulation. They found that at the same temperature, the thermal conductivity of Nafion 117 increases with the increase of water content. In the case of the same water content, the thermal conductivity decreases with the increase of temperature. In the work of Devanathan and Dupuis [23], MD simulations were performed to analyze the effects of length of side chain on the nanostructure of perfluorosulfonic acid membrane, H_2_O clustering, and the diffusion of H_3_O^+^ and H_2_O. Compared with Nafion, the shortened side-chain of PFSA membranes did not appreciably change the structural or dynamic properties of PFSA membranes, while increasing the temperature of the SSC membrane resulted in a much bigger increase in the proton and water diffusion coefficient than changing the side chain length. Cheng and Hong [24] studied the diffusion of water molecules and hydronium ions in Nafion membrane with the fixed water content at 353 K, using MD simulation. The results show that continuous migration and non-continuous hopping constitute the diffusion phenomenon of hydronium ions together. The evaluated self-diffusion coefficients of the hydronium ions and the water molecules are of the same order of magnitude as the experimental results [25]. In the work of Li et al. [26], the classic MD simulation is used to determine the diffusion of water through the Nafion membrane, and the non-equilibrium MD simulation to investigate the interfacial transport. The results show that the interfacial transport coefficients are less sensitive to water content than the self-diffusion coefficients. Compared with the diffusion resistance, the interfacial resistance can be neglected. There are also many atomic-level investigations focused on the effects of temperature [27,28], monomer numbers [29], solvation [30], different chemical groups [31,32] and side chains [33,34,35] on the nanophase-segregated structures and transport properties in different PEMs.

Although the hydrated polyelectrolyte membranes have been extensively studied using molecular dynamics simulations, the systematic research on the effects of hydration and temperature on the microstructure and transport properties of polyelectrolyte membranes is relatively rare. In the present work, molecular dynamics simulations on Nafion 117 system are performed at different hydration and temperature to investigate the effects on the microstructure and the mobility of the water molecules and hydronium ions, the simulation results are compared with relevant experimental and theoretical investigations.

## 2. Materials and Methods

### 2.1. Atomistic Models and Amorphous Cell Construction

**Figure 1:**
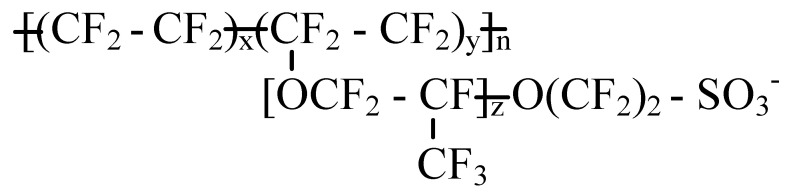
Chemical structure of Nafion.

**Figure 2:**
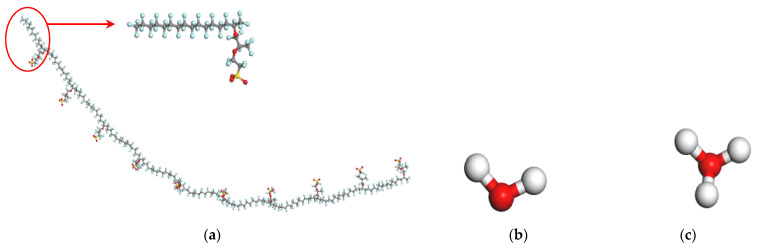
All-atom models of (**a**) Nafion chain, (**b**) water molecular and (**c**) hydronium ion, the white, red, blue, gray, and yellow beads represent hydrogen, oxygen, fluorine, carbon and sulfur atoms, respectively.

**Figure 3:**
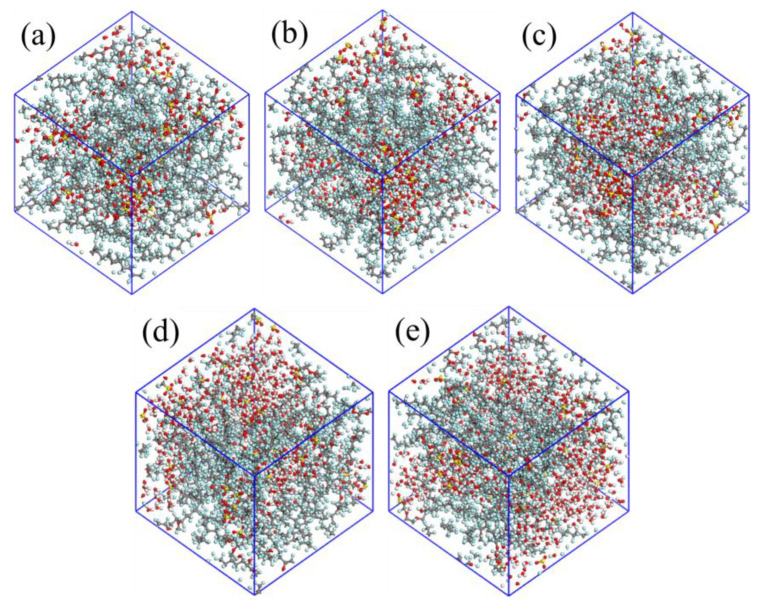
All-atom models of Nafion membranes for different hydration levels, (**a**–**e**) water content λ = 3.5, 7, 10, 13, and 16, respectively.

The chemical structure of Nafion [36] is shown in Figure 1, for Nafion 117, *x* = 7 and *y* = *z* = 1. In this work, each Nafion chain consisted of 10 monomers, so the *n* in Figure 1 is 10. Figure 2a–c are the structure models of Nafion chain, water molecule and hydronium ion, respectively. In order to examine the influence of hydration upon the characteristics of the Nafion membranes, the cell models for water content λ = 3.5, 7, 10, 13 and 16 are constructed, where λ is the ratio of the sum of the number of water molecules and that of hydronium ions over the number of sulfonic groups [37]. Each cell model consists of four Nafion chains and 40 hydronium ions to keep the cell neutral in charge. The number of water molecules in each cell depends on the values of λ. Table 1 outlines the detailed composition of the cell models. The constructed cells for different water contents are displayed in Figure 3a–e.

### 2.2. Force Field

In this work, all the simulations were performed with the COMPASS II (Condensed-phase Optimized Molecular Potentials for Atomistic Simulation Studies) force field [38,39,40]. It is the first ab initio force field that enables accurate and simultaneous prediction of gas-phase properties (structural, conformational, vibrational, and so on) and condensed-phase properties (equation of state, cohesive energies, and so on) for a broad range of molecules and polymers. It is also the first high-quality force field to consolidate parameters of organic and inorganic materials. The function forms can be divided into two categories: valence terms and non-bond interaction terms (Equation (1)). The valence terms are composed of diagonal and off-diagonal cross-coupling terms (Equation (2)), which are the bond-stretching term (*E*_*b*_), angle-bending term (*E*_*θ*_), torsion angle term (*E*_*ϕ*_) and out-of-plane bending term (*E*_χ_), cross term (*E*_*cross*_). The non-bond interactions including the van der Waals (vdW) term (Equation (3)) and the Coulombic term (Equation (4)) are used to describe the interactions between pairs of atoms that are separated by two or more intervening atoms or those that belong to different molecules.

(1) $$E_{total}=E_{valence}+E_{nonbond}$$

(2) $$\begin{array}{ll} E_{valence} & =E_{b}+E_{q}+E_{f}+E_{c}+E_{cross}\\ & =\sum_{b}\left[k_{2}{\left(b-b_{0}\right)}^{2}+k_{3}{\left(b-b_{0}\right)}^{3}+k_{4}{\left(b-b_{0}\right)}^{4}\right]\\ & +\;\sum_{\theta }\left[k_{2}{\left(\theta -\theta_{0}\right)}^{2}+k_{3}{\left(\theta -\theta_{0}\right)}^{3}+k_{4}{\left(\theta -\theta_{0}\right)}^{4}\right]\\ & +\sum_{\phi }\left[k_{1}(1-\cos\phi )+k_{2}(1-\cos2\phi )+k_{3}(1-\cos3\phi )\right]+\sum_{\chi }k_{2}\chi^{2}+\sum_{b,b^{\prime }}k(b-b_{0})(b^{\prime }-b^{\prime }{}_{0})\\ & +\sum_{b,\theta }k(b-b_{0})(\theta -\theta_{0})+\sum_{b,\phi }(b-b_{0})\left[k_{1}\cos\phi +k_{2}\cos(2\phi )+k_{3}\cos(3\phi )\right]\\ & +\sum_{\theta ,\phi }(\theta -\theta_{0})\left[k_{1}\cos\phi +k_{2}\cos(2\phi )+k_{3}\cos(3\phi )\right]+\sum_{b,\theta }k(\theta^{\prime }-\theta^{\prime }{}_{0})(\theta -\theta_{0})\\ & +\sum_{\theta ,\theta ,\phi }k(\theta^{\prime }-\theta^{\prime }{}_{0})(\theta -\theta_{0})\cos\phi \end{array}$$

(3) $$E_{vdW}=\sum_{i,j}\varepsilon_{ij}\left[2{\left(\frac{r^{0}{}_{ij}}{r_{ij}}\right)}^{9}-3{\left(\frac{r^{0}{}_{ij}}{r_{ij}}\right)}^{6}\right]$$

(4) $$E_{coulombic}=\sum_{i,j}\frac{q_{i}q_{j}}{r_{ij}}$$

### 2.3. Molecular Dynamic Simulation

The initial cells are first optimized to minimize their energies by means of the steepest descent method and the conjugate gradients minimization algorithms. After the optimization process, the following dynamics simulation procedures are performed:

(a) MD simulation for 100 ps at 300 K in NVT ensemble.
(b) MD simulation for 100 ps at 300 K in NPT ensemble.
(c) The final structure of NPT MD simulation is heated from 300 K to 600 K, MD simulation is performed for 50 ps for every 100 K increase in temperature in the NVT ensemble.
(d) The final structure of procedure (c) is cooled from 600 K to 300 K, MD simulation is performed for 50 ps for every 100 K decrease in temperature in NVT ensemble.
(e) MD simulation for 100 ps at 300 K in NPT ensemble.

After repeating procedure (c) to (e) three times, the final structure was taken as the initial configuration for equilibrating procedures. Equilibrations using this configuration were carried out for 1 ns at 298 K, 323 K, and 353 K respectively, in the NPT ensemble. The equilibrating procedure was followed by 1ns NVE dynamic simulation at 298 K, 323 K, and 353 K, respectively, from which the trajectories were used for analyzing structural properties and dynamic properties. In the molecular dynamics simulation using the NVE ensemble, the energy of the system is conserved. This is very important and extremely necessary for the diffusion simulation. However, simulations in such an ensemble are not temperature controlled. The monitored, fluctuating temperatures in our NVE simulations for λ = 16 are shown in Figure S1. It can be seen that the temperatures fluctuate up and down around the target temperatures, while the fluctuation is slight.

All MD simulations in this work were performed using Materials Studio 2017 program (Accelrys Inc., San Diego, CA, USA), three-dimensional periodic boundary conditions were applied, and time steps of 1 fs were used. Nose–Hoover thermostats and Berendsen barostats were used to control the temperature and pressure at the desired temperature and ambient pressure for all ensembles. The Ewald summation routine was used to calculate long-range electrostatic forces.

## 3. Results and Discussion

### 3.1. Density

Fully equilibrating the simulated cell is one of the crucial steps in any MD study. During the NPT simulations of equilibrating procedure in this work, the cell density was fluctuant. When the density remained almost unchanged and the fluctuation was slight, it indicated that the system was fully equilibrated. The densities in the course of the equilibration procedures are shown in Figure S2 (λ = 13 for example). It can be seen that the fluctuations of densities are quite slight, less than 3% from 400 ps to 1000 ps, suggesting that the cells are fully equilibrated.

**Figure 4:**
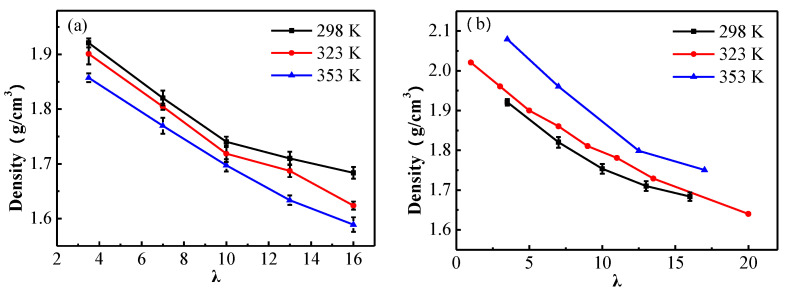
(**a**) The densities of the simulated Nafion membranes for λ = 3.5, 7, 10, 13 and 16 at 298 K, 323 K and 353 K, respectively; (**b**) the comparison of the simulated results with the experimental and theoretical results.

Figure 4a shows the densities of the fully equilibrated cells at the end of the equilibration procedures at different temperatures, and Figure 4b the comparison of the simulated results in this work with the experimental and computational data in literature. From Figure 4a it can be seen that the density gradually decreases with the increased λ at the same temperature, this can be attributed to the swelling of the membrane due to the increasing hydration. The membrane density is lower at higher temperature for the same hydration level, this is due to the thermal expansion of the membrane. Figure 4b shows our simulated densities of membranes at 298 K for different water content, the MD predicted results of Chen [18], and the experimental data measured by Mirris and Sun [7]. Obviously, our computed densities agree well with the experimental and the theoretical results.

### 3.2. Radial Distribution Functions and Coordination Numbers

**Figure 5:**
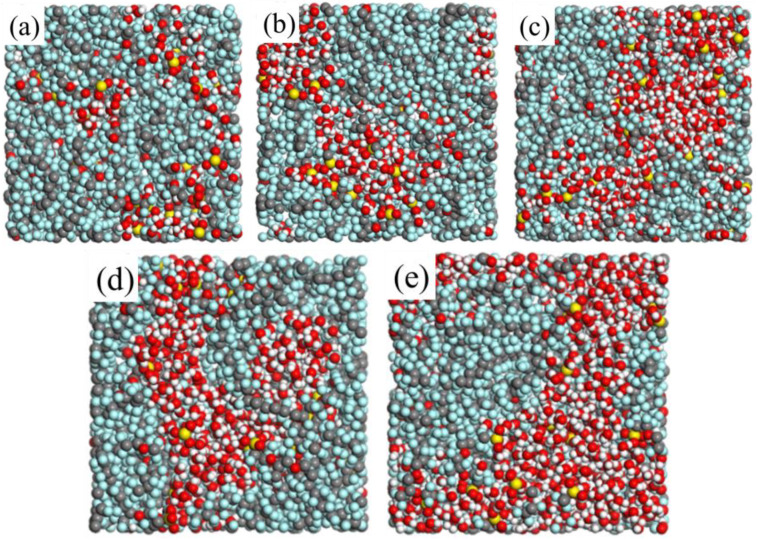
The snapshots of the simulated Nafion membranes (**a**–**e**) for λ = 3.5, 7, 10, 13 and 16 at the end of the MD simulations at 298 K.

In order to understand the microstructure of the hydrated membranes, it is necessary to look into the configurations obtained from the MD simulations. The snapshots of each system for λ = 3.5, 7, 10, 13 and 16 at the end of the simulations at 298 K are shown in Figure 5. The snapshots clearly show that the hydrophilic regions are constituted by the water molecules, hydronium ions and the sulfonate groups, and the hydrophobic regions are constituted by the backbones of the Nafion polymer. In order to qualitatively study the interactions among the sulfonate groups, hydronium ions, and water molecules, several radial distribution functions (RDFs) between different atoms in the hydrated Nafion systems are calculated.

The RDF is defined as a measure of the probability of finding an atom B at a distance *r* from a reference atom A, and can be calculated by Equation (5) [41].

(5) $$g_{A-B}(r)=\frac{Vn_{B}}{4N_{B}\pi r^{2}dr}$$

where *n*_*B*_ is the number of B atoms situated at a distance *r* in a shell of thickness *d*_*r*_ from particle A, *N*_*B*_ and *V* represent the number of B atoms in the system and the total volume of the system, respectively. The coordination numbers (CNs) are obtained by integrating the area under the corresponding RDFs to evaluate the correlation of different molecules or groups quantitatively [42].

**Figure 6:**
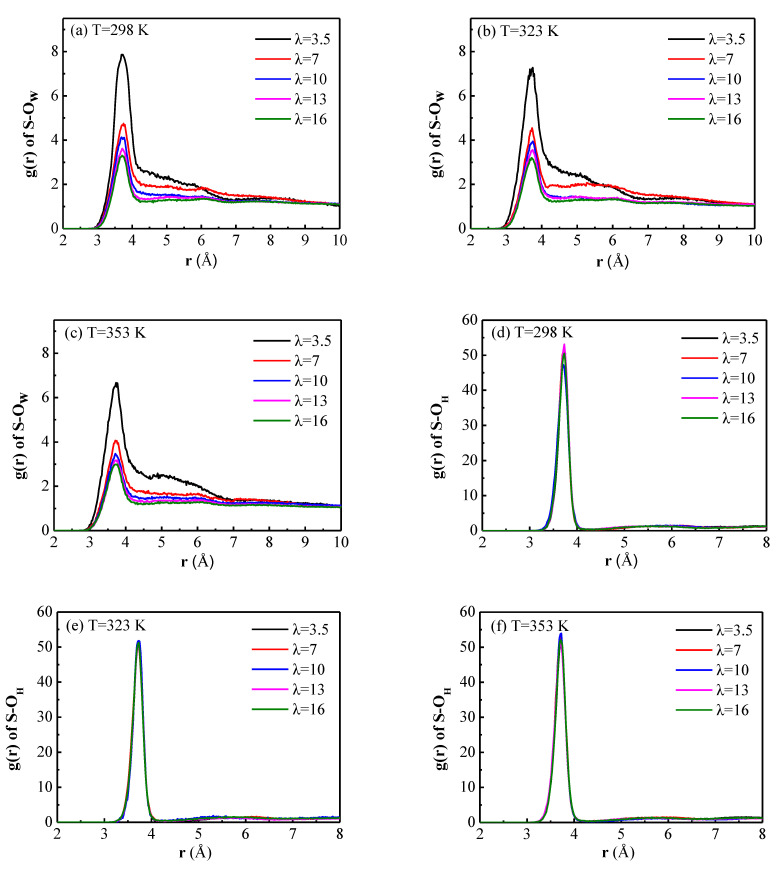
The radial distribution functions (RDFs) of (**a**–**c**) S-O_w_ and (**d**–**f**) S-O_H_ for water content λ = 3.5, 7, 10, 13, and 16 at 298 K, 323 K and 353 K, respectively.

The RDFs of S-O_w_ (RDFs between sulfur atoms of sulfonate groups and oxygen atoms of water molecules) for different water contents at 298 K, 323 K and 353 K are shown in Figure 6a–c, respectively. For all S-O_w_ RDFs, the intense peaks occurred at about 3.72 Å, which means there is a hydration shell around the –SO_3_^−^ at a distance of 3.72 Å from the sulfur atom. This is consistent with the previous findings [12,15,16]. When the water content increases, the peak intensity decreases, suggesting that the interaction between water molecules and side chains decreases with the increasing hydration. It is interesting to find that there is a sudden drop for the peak intensity, with the water content increasing from 3.5 to 7. However, with the water content increasing from 7 to 16, the peak intensity is slightly reduced.

The interactions of −SO_3_^-^ and hydronium ions were studied by the RDFs of S-O_H_ (RDFs between sulfur atoms of sulfonate groups and oxygen atoms of hydronium ions) displayed in Figure 6d–f. The peak positions are extremely similar to those of S-O_w_ RDFs, at about 3.72 Å, proving that the sulfonate groups attract both water and hydronium ions. However, the heights of S-O_H_ RDFs are much greater than those of S-O_w_ RDFs, and not that sensitive to the water content, which is attributed to the strongly mutual attraction between the positively charged hydronium ions and the negatively charged sulfonate groups.

The CNs of water molecules and hydronium ions around the sulfur atoms are shown in Table 2. It can be seen that as the degree of hydration increases, the CN of water molecules increases, while the CN of hydronium ions decreases. This is due to the solvation of sulfonate groups and hydronium ions by water molecules. When there are more water molecules competing for the attention of the sulfonate groups, the attraction between the sulfonate groups and the hydronium ions seems to be weakened. The sum of the CNs of water molecules and hydronium ions increases with the increasing water content at the same temperature. In other words, as the hydration level increases, the size of water clusters inside the membrane increases, and a connected transmission channel is formed. Increasing the temperature can slightly reduce the CN of water molecules, however, the CN of hydronium ions is increased. While the sum of the CNs of water molecules and hydronium ions is reduced with the increasing temperature for the same water content, this is probably due to the enhanced mobility of water at a higher temperature. Comparing the CNs of water molecules and hydronium ions, the result of Tse [33] is conformed, the higher the CN of water molecules, the lower the CN of hydronium ions. Thus the competition between water molecules and hydronium ions for the attraction of the sulfonate groups is also verified.

### 3.3. Sizes and Connectivity of Water Clusters

The cursory visual inspection of the snapshots in Figure 5 suggests that the water clusters are quite small at λ = 3.5, and the connectivity between the clusters is poor. As the water content increases from 3.5 to 16, the cluster size increases, and the connected channels exist between the clusters. This is very important for the transportation of water molecules and hydronium ions in the membrane and for them not to be trapped in isolated pores. In order to demonstrate the effects of hydration level and temperature on the sizes and the connectivity of clusters qualitatively, the water clusters were characterized with a cutoff radius of 3.5 Å, and the cluster size distributions were clarified. If the distance between the oxygen atoms of two water molecules (including the hydronium ions) is determined to be smaller than 3.5 Å, these two molecules are deemed to belong to the same cluster. Then we search the water molecules and hydronium ions around these molecules with the cutoff radius and continue until all clusters in the system are found.

**Figure 7:**
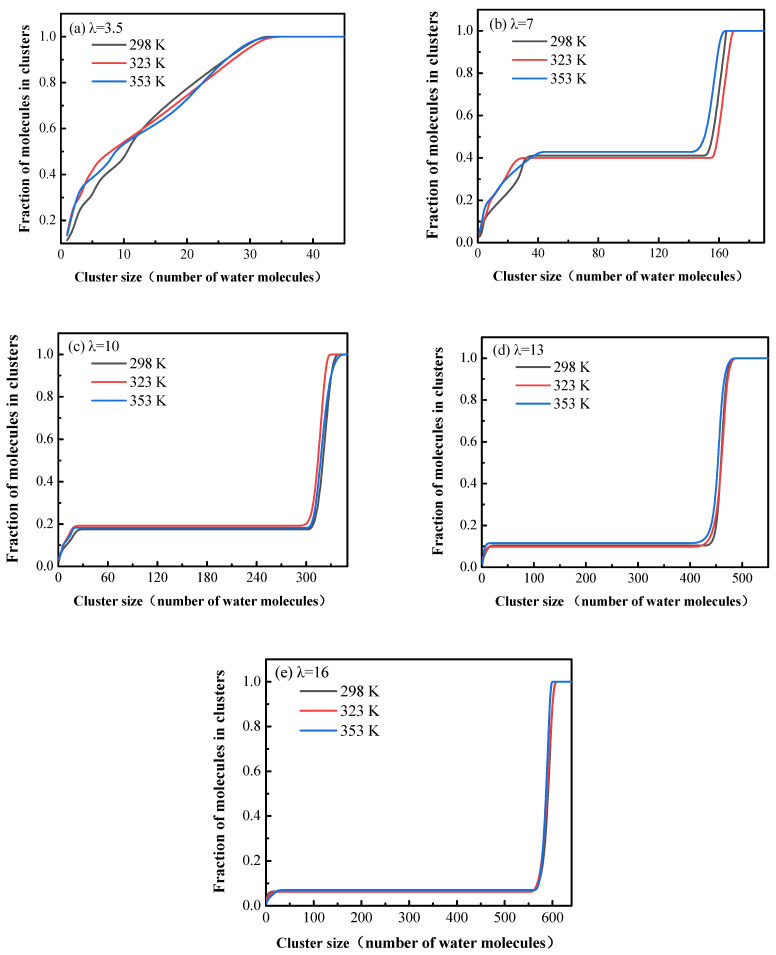
Cumulative fraction of the number of molecules in clusters at different temperatures for different hydration levels, (**a**–**e**): λ = 3.5, 7, 10, 13 and 16, respectively.

Figure 7 displays the cumulative fraction of the number of molecules in clusters for different hydration levels at different temperatures. It can be seen that the temperature has no obvious influence on the trend of the cumulative fraction, but the hydration level does. The curves for λ = 3.5 continuously rise until the fractions reach 1 at a cluster size of about 30 molecules. This indicates that all the water molecules and hydronium ions are dispersed in many small clusters that are not connected to each other. When the water content increases to 7, the behavior of the curves is quite different. The cumulative fractions rise to about 0.4 at about 40, then are constant at an extended region, and rapidly rise to 1 at around 160, suggesting that about 40% of the water molecules and hydronium ions are found in some small clusters with the sizes not bigger than 40, while the other 60% form one large cluster. As the hydration level becomes higher (λ = 10 and 13), the flat areas of the curves become lower and lower, which indicates that there are fewer and fewer molecules in the small clusters, and more and more water molecules and hydronium ions join the large cluster. In other words, as the hydration level increases, the clusters become bigger until they connect with each other to become a larger cluster. For the highest hydration level (λ = 16), more than 90% of the water molecules and hydronium ions are in a large cluster to form a hydrophilic channel spanning the entire system.

The analysis of the cumulative fraction of the number of molecules in clusters confirms the conclusion of cursory visual inspection on the microstructure of the hydrated membrane, that the phase separation of the membrane is affected a lot by the hydration level. As the water content increases, the sizes of water clusters in the system increase, and the connection between clusters is improved, forming the continuous channels for transportation of water molecules and hydronium ions.

### 3.4. Mean Square Displacements and Diffusion Coefficients

The transport of water and hydronium are crucial for a polyelectrolyte membrane. In the classical MD simulations, the self-diffusion coefficients can be estimated by studying the mean square displacements (MSDs) [43,44]. The mean square displacement is defined as follows:

(6) $$MSD(t)=\frac{1}{N}\langle \sum_{i=1}^{N}|r_{i}(t)-{r_{i}(0)|}^{2}\rangle$$

where *r*_*i*_ (*t*) is the location of atom *i* at *t* time, *N* is the number of freely diffusing atoms in the system.

According to the Einstein’s diffusion law, the self-diffusion coefficient *D* can be calculated by Equation (7).

(7) $$D=\frac{1}{6N}\underset{t\to \infty }{\lim}\frac{d}{dt}\sum_{i=1}^{N}\langle {\left[r_{i}(t)-r_{0}(t)\right]}^{2}\rangle$$

If we define the slope of the curve of MSD with time as *a*, according to Equations (6) and (7) the self-diffusion coefficient *D* can be easily estimated as:

(8) $$D=\frac{a}{6}$$

**Figure 8:**
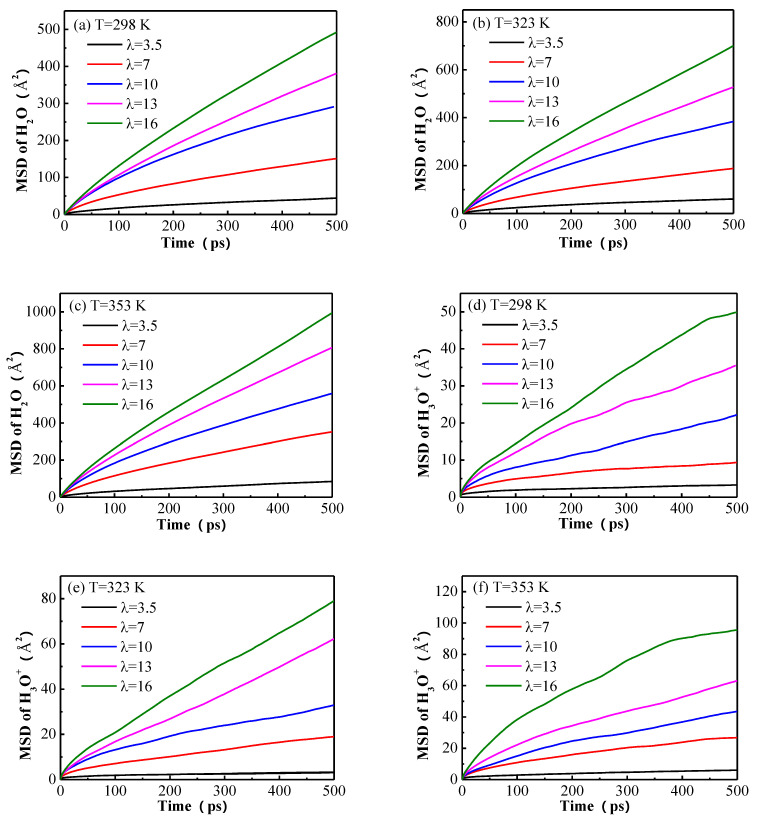
The mean square displacements (MSDs) of (**a**–**c**) water molecules and (**d**–**f**) hydronium ions for water content λ = 3.5, 7, 10, 13, and 16 at 298 K, 323 K and 353 K, respectively.

The MSDs of water molecules and hydronium ions calculated from the final 1 ns of the dynamics simulation trajectories for different hydration levels at different temperatures are displayed in Figure 8. It can be seen that the MSDs of water molecules and hydronium ions both rise with the increase of water content and temperature. The MSD of water molecules is much higher than that of hydronium ions, at the same temperature and hydration level. That is to say, the mobility of water molecules is much stronger than that of hydronium ions, and the influence of temperature and hydration level on the diffusivity of those particles is prominent.

We can also discuss the above phenomenon more precisely in terms of the diffusion coefficients of water molecules and hydronium ions. The diffusion coefficients calculated from the linear regime of the MSD curves are shown in Table 3. In general, the diffusion coefficient of water molecules is much larger than that of hydronium ions. This is a reflection of the electrostatic interaction of the positively charged hydronium ions with the negatively charged sulfonate groups, which is relatively immobile. On the other hand, the effect of hydration level on the mobility of hydronium ions is greater than that of water molecules. For example, the diffusion coefficient of water molecules for λ = 3.5 at 353 K is about 25 times of that of hydronium ions, but for λ = 16, the ratio is reduced to about 10. This is because the increased solvation of the hydronium ions by the water molecules weakens the electrostatic interaction of the hydronium ions with the sulfonate groups, thus the lability of the hydronium ions is increased. Another reason is that the expansion of the membrane by water molecules makes the diffusion channels of hydronium ions larger. Although the diffusion capacity of water molecules in membrane is significantly strengthened by the increasing hydration level, due to the improved connectivity of water channels, it is still much smaller than the self-diffusion coefficient of bulk water, which is because of the polymer blockage. Compared with the self-diffusion coefficient of hydronium ions in bulk water, the diffusion capacity of hydronium ions in the hydrated membrane is smaller. The main reason for the difference is the electrostatic interaction between the hydronium ions and the sulfonate groups in the membrane, and another factor is the numerous hydrogen bonding network that aids efficient proton hopping in bulk water.

**Figure 9:**
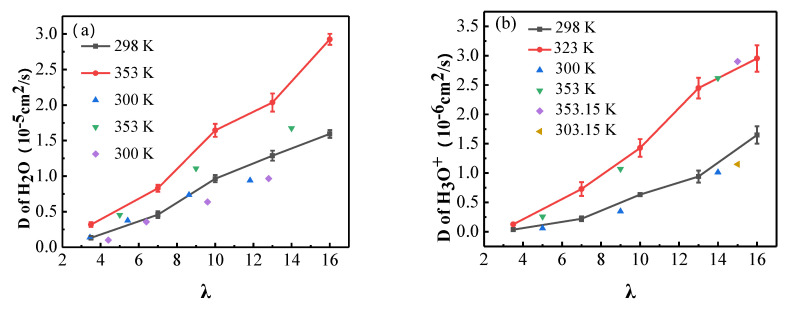
The comparison of simulated diffusion coefficients in the present study with the values in literature: (**a**) water molecules and (**b**) hydronium ions at 298 K and 353 K. Scattered data come from the literature.

From Table 3, it also can be seen that the diffusion coefficients of water molecules and hydronium ions are both significantly increased by the increasing temperature for all hydration levels. This is a reflection of the fact that the higher the temperature, the higher the kinetic energy of molecules, and thus the mobility is strengthened. In addition, the expansion of the diffusion channels in the membrane caused by the increased temperature also contributes to the mobility of water molecules and hydronium ions. Figure 9 shows the comparison of the simulated results in this study with the values in other works. It can be seen that the diffusion coefficients of water molecules and hydronium ions at 298 K and 353 K in this work are in good accord with the reported values [19,20,21,33]. Compared with the experimental results, the calculated diffusion coefficients of water molecules are considerably larger, e.g., the experimental values are 0.6 × 10^−5^ cm^2^/s and 1.25 × 10^−5^ cm^2^/s at 298 K and 353 K for λ = 14 respectively [9], while the simulated results are 1.288 × 10^−5^ cm^2^/s and 2.036 × 10^−5^ cm^2^/s for λ = 13 at the same temperature. This may be due to the periodic boundary conditions applied in our simulation, which is just an ideal treatment for an infinite system. The experimental results were obtained by describing the diffusion processes in a restricted geometry with ill-defined boundaries. However, the simulated diffusion coefficients of hydronium ions are significantly smaller than the experimental ones, e.g. the simulated results are 1.649 × 10^−6^ cm^2^/s at 298 K and 2.953 × 10^−6^ cm^2^/s at 353 K for λ = 16, while the experimental values are 7 × 10^−6^ cm^2^/s and 10 × 10^−6^ cm^2^/s at 303.15 K and 353.15 K, respectively [8]. This is because the structural diffusion via the Grotthuss mechanism cannot be described by classical MD simulations, and only the vehicular transport mechanism is applied to characterize the diffusion of the protons in the hydrated membranes in the present work. The same difference between the computed diffusion coefficients and the experimental data was also reported by Venkatnathan [27] and Cui [20]. This is also a reflection of the competition between the water molecules and the hydronium ions in the classical MD simulations.

## 4. Conclusions

Molecular dynamics simulations were performed on Nafion 117 polymer electrolyte membrane for λ = 3.5, 7, 10, 13, 16 at 298 K, 323 K and 353 K, to determine the effects of temperature and hydration on the microstructure and transport properties of a polyelectrolyte membrane. The simulated results of the densities of the membrane at 298 K agree well with the experimental and theoretical results. The density gradually decreases with the increase of water content at the same temperature, and this can be attributed to the swelling of the membrane due to the increasing hydration level. For the same hydration level, the density decreases with the increase of temperature, and this is a reflection of the thermal expansion of the membrane.

For all hydration levels, the first peaks of RDFs of S-O_w_ and S-O_H_ appear at about 3.72 Å, the difference is that the heights of the first peaks of S-O_H_ RDFs are much larger than those of S-O_w_ RDFs, suggesting that the interaction of hydronium ions and the sulfonate groups is much stronger. The CNs of water molecules and hydronium ions around the sulfonate groups in the first solvation shell are obtained by integrating the area under the corresponding RDFs. Comparing the CNs of water molecules and hydronium ions for the different membranes at the same temperature or for the same membrane at different temperatures, it can be seen that when the CN of water molecules is higher, the CN of hydronium ions is lower, vice versa. There seems to be a competition between water molecules and hydronium ions for the space close to sulfonate groups.

Water clusters are characterized with a cutoff radius of 3.5 Å, and all of the clusters are found in the simulated membranes. The analysis of the cumulative fraction of the number of molecules in clusters suggests that the phase separation of the membrane depends a lot on hydration. However, the influence of temperature on the structure is found to be insignificant. As the hydration level increases from λ = 3.5 to 16, the clusters become bigger until they connect with each other to become a larger cluster spanning the entire system. This is consistent with the results of the cursory visual inspection of the snapshots of the simulated membranes.

The diffusion coefficients of water molecules and hydronium ions are calculated according to the MSDs. Because of the strong electrostatic interaction of the hydronium ions with the sulfonate groups, the diffusion coefficient of hydronium ions is always much smaller than that of water molecules, when compared at the same temperature and hydration level. The mobility of water molecules and hydronium ions are both strengthened by the increased hydration level, however, the mobility of hydronium ions is more sensitive. Increasing the temperature also promotes the diffusion of water molecules and hydronium ions. Due to the polymer blockage and the electrostatic interaction between the hydronium ions and the sulfonate groups, the calculated diffusion coefficients of water molecules and hydronium ions in hydrated membranes are quite smaller that in bulk water

The calculated diffusion coefficients in the present study compare well with those reported in other works; however, they differ from the experimental data. The diffusion coefficients of water molecules are larger than the experimental values, while those of hydronium ions are smaller. This is due to the unavoidable disadvantages of MD simulation, and the same trend has also been reported in literature.

## Figures and Tables

**Table 1:** Composition of the hydrated Nafion membranes.

λ	No. of Nafion Chains	No. of Water Molecules	No. of Hydronium Ions
3.5	4	100	40
7	4	240	40
10	4	360	40
13	4	480	40
16	4	600	40

**Table 2:** The coordination numbers (CNs) of water molecules and hydronium ions around the sulfur atoms with a cutoff of 4.6 Å and 4.25 Å, respectively.

λ	CNs of Water(0–4.6 Å)			CNs of Hydronium Ion(0–4.25 Å)		
	298 K	323 K	353 K	298 K	323 K	353 K
3.5	2.457 ± 0.075	2.375 ± 0.072	2.277 ± 0.082	2.316 ± 0.039	2.368 ± 0.054	2.387 ± 0.042
7	3.723 ± 0.078	3.504 ± 0.098	3.261 ± 0.066	2.028 ± 0.006	2.112 ± 0.013	2.206 ± 0.039
10	3.952 ± 0.085	3.866 ± 0.084	3.586 ± 0.078	1.888 ± 0.005	1.926 ± 0.007	2.005 ± 0.035
13	4.612 ± 0.080	4.477 ± 0.069	4.208 ± 0.069	1.749 ± 0.032	1.786 ± 0.057	1.862 ± 0.048
16	5.050 ± 0.074	4.872 ± 0.075	4.588 ± 0.066	1.570 ± 0.065	1.621 ± 0.025	1.654 ± 0.060

**Table 3:** The calculated diffusion coefficients of water molecules and hydronium ions for water content λ = 3.5, 7, 10, 13, and 16 at 298 K, 323 K and 353 K.

λ	D of H_2_O (×10^−5^ cm^2^/s)			D of H_3_O^+^ (×10^−6^ cm^2^/s)		
	298 K	323 K	353 K	298 K	323 K	353 K
3.5	0.132 ± 0.026	0.173 ± 0.028	0.319 ± 0.034	0.035 ± 0.014	0.085 ± 0.013	0.125 ± 0.018
7	0.458 ± 0.047	0.612 ± 0.061	0.829 ± 0.048	0.221 ± 0.041	0.435 ± 0.092	0.728 ± 0.118
10	0.964 ± 0.051	1.255 ± 0.070	1.645 ± 0.092	0.632 ± 0.021	0.826 ± 0.071	1.428 ± 0.151
13	1.288 ± 0.071	1.680 ± 0.055	2.036 ± 0.117	0.941 ± 0.103	1.821 ± 0.079	2.449 ± 0.177
16	1.594 ± 0.054	2.166 ± 0.043	2.925 ± 0.077	1.649 ± 0.149	2.449 ± 0.134	2.953 ± 0.226
Bulk	2.299 [45]	3.983 [45]	6.582 [46]	93.1 [47]		

## Supplementary Materials

The following are available online at https://www.mdpi.com/article/10.3390/membranes11090695/s1, Figure S1: Monitored temperatures in NVE simulations for λ = 16. Figure S2: The densities in the course of the equilibration procedures for λ = 13.
