# Supplementary material for: Effects of Hydration and Temperature on the Microstructure and Transport Properties of Nafion Polyelectrolyte Membrane: A Molecular Dynamics Simulation
Source: Membranes (Basel). 2021 Sep 8;11(9):695. doi: 10.3390/membranes11090695 (PMC8467011; doi:10.3390/membranes11090695)
Supplement: Supplementary file 1 [file membranes-11-00695-s001.zip › membranes-1346251-supplementary.pdf]

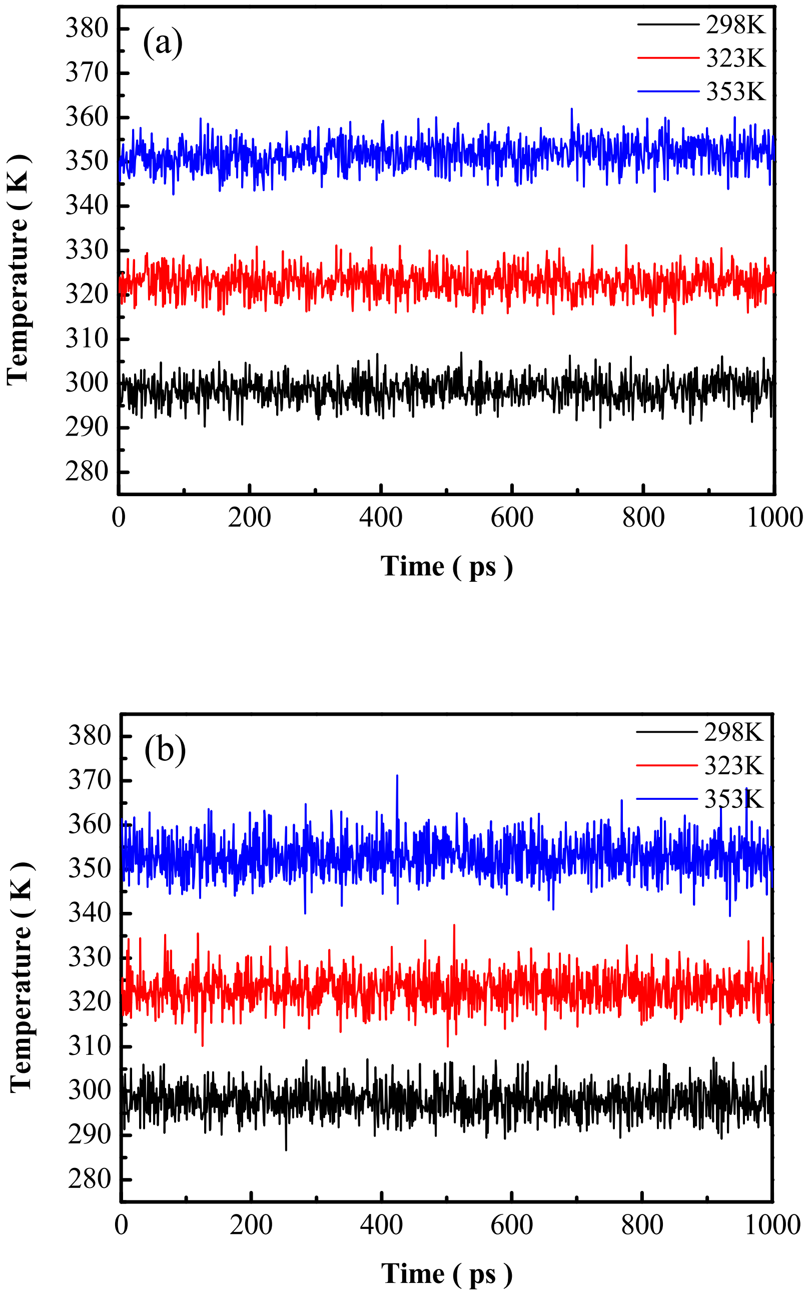

Figure S1. Monitored temperatures in NVE simulations for λ=16.

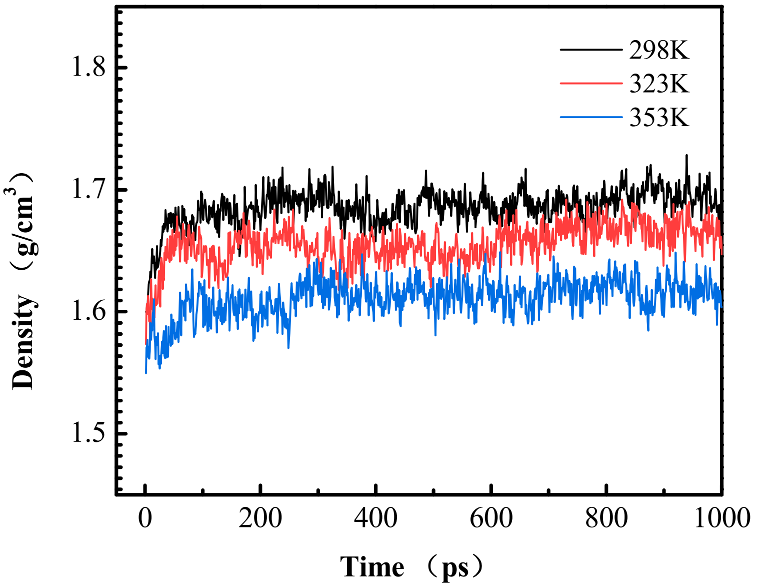

Figure S2. The densities in the course of the equilibration procedures for λ=13.
